# Exploring the potential association between dietary factors and autism spectrum disorder: a Mendelian randomization analysis and retrospective study

**DOI:** 10.3389/fnut.2025.1716044

**Published:** 2026-01-12

**Authors:** Yan-Shuo Guo, Yu Wang, Meng-Na Zhu, Chao Che, Xiao-Xiao Yu, Zhi-Feng Cai, Juan Leng, Kun-Ping Chen, Ai-Hua Cao

**Affiliations:** 1 Department of Paediatrics, Shandong University Qilu Hospital, Jinan, Shandong, China; 2 Department of General Surgery, Qilu Hospital of Shandong University Dezhou Hospital, Dezhou, Shandong, China

**Keywords:** autism spectrum disorder, dietary factors, retrospective study, Mendelian randomization, mediation analysis

## Abstract

**Background:**

Autism spectrum disorder (ASD) is a neurodevelopmental condition involving complex genetic and environmental interactions. This study aimed to investigate the causal relationships between dietary factors, ketones, food allergy, and the risk of ASD using Mendelian randomization (MR) and clinical research.

**Methods:**

We used two-sample MR to analyze the causal associations between 199 dietary factors, ketones, food allergy, and ASD risk using genome-wide association study (GWAS) data. Validity was assessed using sensitivity analyses. Additionally, a retrospective study (*n* = 78, age range 2–7 years) evaluated the clinical effects of a gluten-free casein-free (GFCF) diet on ASD symptoms, as measured by the Childhood Autism Rating Scale (CARS) and the Autism Diagnostic Observation Schedule, Second Edition (ADOS-2).

**Results:**

MR analysis identified significant positive causal effects on ASD risk for wholemeal pasta (OR: 16.0, 95% CI 2.86–89.4, *p* = 0.002) and cheese spread (OR: 9.53, 95% CI 1.64–55.4, *p* = 0.020). It is crucial to emphasize that these estimates represent the lifetime effect of a genetic predisposition to a higher intake level, not the risk from short-term consumption. The very wide confidence intervals indicate substantial uncertainty in the point estimates. Banana had a protective effect (OR: 0.50, 95% CI: 0.30–0.83, *p* = 0.008). No causal links were identified for the other factors. Mediation analysis suggested that cheese spread intake increased ASD risk partly by lowering HLA-DR + T cell CD45 levels (10.2% mediation) and increasing anti-Epstein–Barr virus IgG seropositivity (12.9% mediation). Clinically, Although the GFCF diet did not significantly improve ADOS-2 and CARS scores, it showed greater improvement compared to the normal diet group. This diet significantly reduced milk- and wheat-specific IgG levels, indicating its ability to effectively modulate immune responses.

**Conclusion:**

This study provides genetic evidence of causal relationships between specific dietary factors and ASD risk. Clinical data indicate that adhering to a gluten-free, casein-free diet and avoiding related allergenic foods can effectively modulate food-specific immune responses and may also improve ASD symptoms. These findings contribute to deepening our understanding of ASD etiology and optimizing nutritional treatment protocols.

## 1 Introduction

Autism Spectrum Disorder (ASD) is a neurodevelopmental disorder characterized by core deficits in social communication and interaction, along with the presence of restricted, repetitive patterns of behavior and interests (1). It is commonly comorbid with intellectual disabilities (2), immune system dysregulation (3), and inflammatory gastrointestinal problems (4). ASD’s development is understood as a multifactorial process, involving complex interactions between adverse prenatal environments, childhood lifestyle factors, genetic predispositions, and immune, inflammatory, and psychosocial influences (4–8). Currently, there is no definitive clinical treatment for ASD, and interventions primarily target educational, rehabilitative, pharmacological, and psychotherapeutic approaches to improve emotional and behavioral issues (9). However, despite early intensive interventions, improvements in certain symptoms remain limited (10).

Children with ASD frequently experience a range of gastrointestinal disorders (GIDs). Combined with their common communication difficulties, this suggests that certain behavioral core symptoms may reflect underlying abdominal pain or discomfort (10–14). This has led to increased interest in dietary interventions as potential treatments for ASD.

Dietary interventions for ASD typically include a gluten-free casein-free (GFCF) diet, elimination of harmful food sensitivities, and a ketogenic diet, all of which have been shown to improve certain symptoms of autism. The GFCF diet eliminates gluten-containing cereals (such as wheat, rye, barley, and oats) and casein from dairy products such as milk, butter, and cheese (15, 16). The GFCF diet was selected as the primary focus of our clinical investigation due to its prominent role in existing observational literature reporting symptomatic improvements in ASD and its proposed pathophysiological rationale involving the gut-brain axis and immune responses. In contrast, the ketogenic diet is low in carbohydrates, high in fat, and moderate in protein, and aims to induce a metabolic state similar to that of fasting. In this state, ketone bodies produced from the metabolism of stored fats replace glucose as the primary energy source for the brain. The ketogenic diet is widely used to treat refractory epilepsy and metabolic syndrome and has been effective in controlling epilepsy-related behavioral abnormalities in autism (17). It also exerts neuroprotective and antioxidant effects by improving mitochondrial bioenergetics and neurotransmitter regulation and reducing neuroinflammation and oxidative stress (18). Ketone bodies, particularly 3-hydroxybutyrate (3-HB), play a central role in regulating inflammation and oxidative stress via complex molecular signaling pathways (19, 20). Evidence suggests that mild ketosis enhances metabolism, extends lifespan, and alleviates neurological disorders (18, 21).

Observational studies support the idea that the GFCF diet, elimination diets, and ketogenic therapies can improve the behavioral symptoms of children with ASD (22–26). A growing body of literature indicates that diet plays a crucial role in regulating ASD through its effects on inflammation, immune response, gut microbiota, and antibody levels. Understanding whether dietary changes are beneficial is crucial for both clinicians and patients with ASD.

Furthermore, clinical observations suggest that food intolerance is often present in ASD, with significantly higher levels of milk- and wheat-specific IgG antibodies than those in healthy children (24). Reductions in these IgG antibodies, along with improvements in gastrointestinal symptoms and abnormal behaviors, have been observed in some children on the GFCF diet (25). This suggests that food-specific IgG antibodies, particularly those targeting milk and wheat, may play a role in ASD pathophysiology. The prevailing hypothesis is that increased intestinal permeability in children with ASD allows incompletely digested gluten and casein peptides to enter the bloodstream, triggering immune responses and the production of IgG antibodies (26). These peptides or neurotoxic substances derived from them may cross the blood–brain barrier, interfering with neurological function and contributing to behavioral abnormalities (27).

Given this, our study aimed to investigate the correlation between the GFCF diet, serum milk/wheat-specific IgG levels, and clinical ASD symptoms using a retrospective study. Additionally, we used Mendelian randomization to examine the genetic associations between dietary intake and ASD and explored the inflammatory factors, immune cells, and gut microbiota involved using mediation analyses. This study aimed to provide an evidence-based foundation for dietary management strategies in ASD.

## 2 Materials and methods

### 2.1 Mendelian randomization

#### 2.1.1 Study design

**Figure 1:**
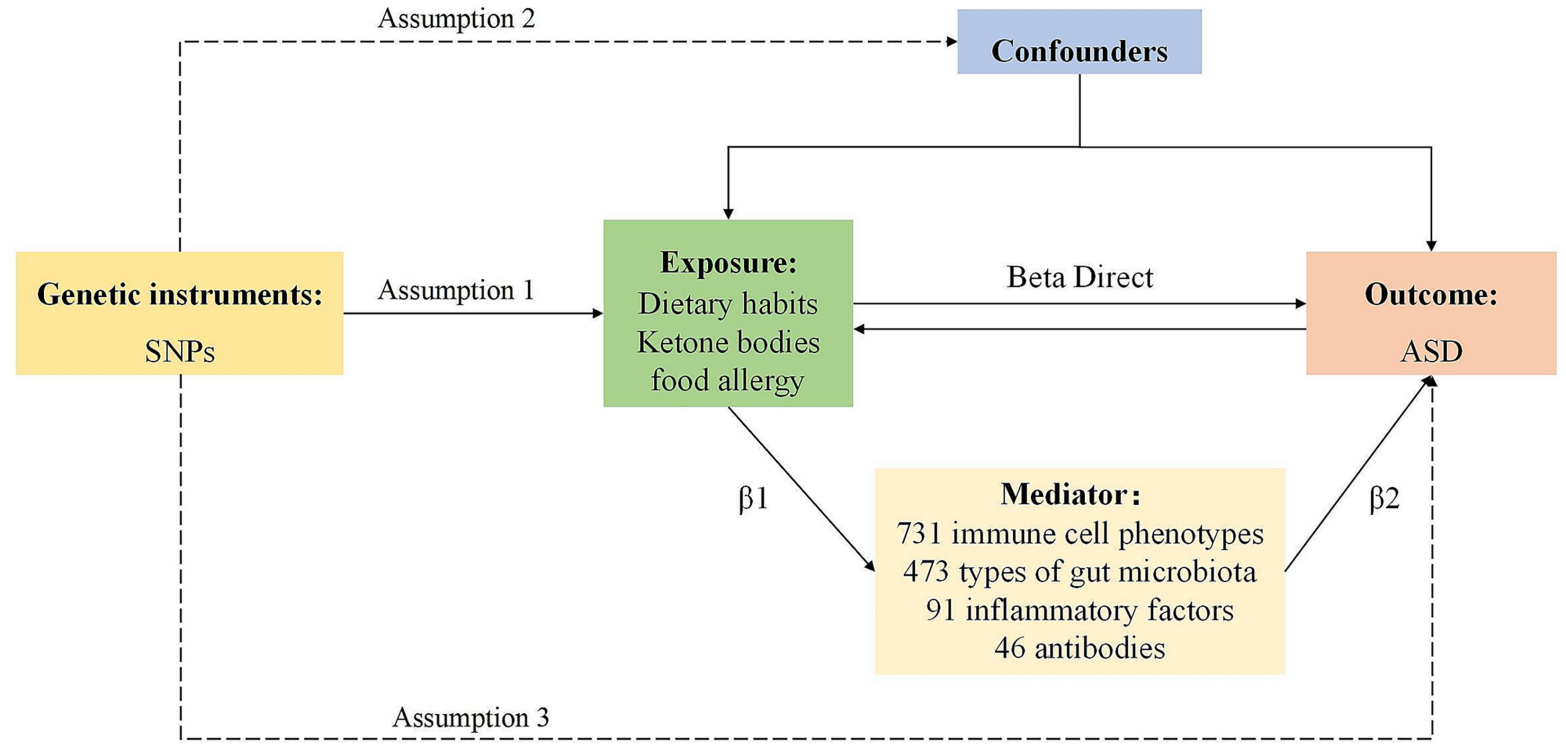
The workflow of this study.

Employing a bidirectional two-sample Mendelian Randomization (MR) framework, this study rigorously evaluated the potential causal interplay between dietary factors, ketone bodies (3-HB and acetoacetate levels), food allergy, and ASD. We employed inverse variance weighting (IVW) to assess the primary causal effects, followed by reverse MR analysis to evaluate potential reverse causality. Additionally, two-step MR and multivariate MR (MVMR) analyses were conducted using inflammatory factors, immune cells, gut microbiota, and antibody immunity as potential mediators to elucidate their roles in the causal pathways. The study design is illustrated in the diagram below (Figure 1). Given the use of publicly available anonymized data, an ethical review was not required for this study.

#### 2.1.2 Data sources

Detailed descriptions of all GWAS data sources are provided in Table 1 and Supplementary Table S1.

**Table 1:** Summary of GWAS data.

Trait	Sample size	Ancestry	PubMed ID or GWAS ID
Exposures			
Banana intake	64,949	European	ukb-b-5362
Wholemeal pasta intake	64,949	European	ukb-b-4150
Cheese spread intake	64,949	European	ukb-b-6185
Other 196 dietary habits		Detailed in the Supplementary Table S1	
3-Hydroxybutyrate levels	113,594	European	ebi-a-GCST90092811
Acetoacetate	115,075	European	met-d-Acetoacetate
Food allergy	493,478	European	finngen_R12_FOOD_ALLERGY
Mediator			
731 immune cell phenotypes		European	GCST0001391 to GCST0002121
473 types of gut microbiota		European	GCST90032270 to GCST90032444
91 inflammatory factors		European	GCST90274848 to GCST90274758
46 antibodies		European	GCST90006884 to GCST90006929
Outcome			
Autism spectrum disorder	46,351	European	ieu-a-1185

#### 2.1.3 Selection of genetic instrumental variables

For the forward MR analysis, dietary factors, ketone body levels, and food allergies were selected as exposure variables. Instrumental variables (IVs) were identified using a two-step process: First, single nucleotide polymorphisms (SNPs) with *p* < 5 × 10^−6^ were selected as potential IVs. This relaxed significance threshold, compared to the conventional genome-wide threshold (p < 5 × 10^−8^), was employed to capture a sufficient number of genetic instruments for dietary traits, which are typically polygenic and have modest heritability. We acknowledge that this approach could potentially increase the risk of weak instrument bias; therefore, we rigorously assessed and reported instrument strength for all exposures. Second, linkage disequilibrium (LD) clumping was performed using the European 1,000 Genomes Project data (*R*^2^ < 0.001, clumping distance = 10,000 kb) to ensure the independence of the selected SNPs. The strength of the instrumental variables was assessed using the F-statistic. The mean F-statistic for all exposure variables exceeded the empirical threshold of 10, thereby largely alleviating concerns about weak instrumental variable bias. A complete list of F-statistics for all 199 dietary exposures is provided in Supplementary Table S9. The validity of each SNP as an IV was assessed using the F statistic, with values greater than 10 indicating strong instrumentality of the SNP. For reverse MR analysis, ASD was used as the exposure variable, and SNPs with *p* < 5 × 10^−8^ were selected as IVs. The same LD clumping parameters were used in this study. In the mediation analysis, 91 circulating inflammatory proteins, 731 immune cells, 473 gut microbiota, and 46 antibody immune responses were considered potential mediators, with a relaxed *p* < 5 × 10^−6^ threshold to include a broader range of SNPs in the analysis.

#### 2.1.4 Mendelian randomization analysis

As the principal analytical strategy, the IVW method was implemented to derive causal effect estimates, effectively controlling for confounding bias under the assumption of no horizontal pleiotropy in the MR analysis framework. The IVW method, combined with Wald ratios in fixed-effects meta-analyses, provides a robust inference of causal relationship. The weighted median approach was used as a complementary method, ensuring consistency even when up to 50% of the data were influenced by the null instruments. MR-Egger regression was employed to adjust for directional pleiotropy, although its results should be interpreted with caution because of lower statistical power. The Wald ratio (WR) method was used when only one SNP passed quality control. Additional methods, such as MR-PRESSO, were applied to enhance the reliability of the results of this study.

#### 2.1.5 Sensitivity analysis

Comprehensive sensitivity analyses were performed to examine the horizontal pleiotropy and heterogeneity. Heterogeneity across instrumental variables was quantified using Cochran’s Q statistic, where *p*-values exceeding 0.05 supported the homogeneity assumptions. Horizontal pleiotropy detection incorporated both MR-Egger intercept analysis and MR-PRESSO global outlier testing, with non-significant findings (*p* > 0.05), indicating the absence of substantial pleiotropic effects. Result stability was verified through iterative leave-one-out sensitivity testing by systematically excluding individual SNPs and recalculating causal estimates.

#### 2.1.6 Mediation analysis

A two-step approach was used to explore the mediating roles of inflammatory factors, immune cells, gut microbiota, and antibody immunity in the causal pathway between dietary factors, ketone bodies, and food allergy in ASD. First, the associations between these potential mediators and ASD were assessed using MR analysis. Subsequently, the correlations between the identified mediators and exposure habits were assessed. The mediating effects were quantified using the delta method, and the proportions of mediation were calculated. The entire process was implemented using the TwoSampleMR R package.

### 2.2 Retrospective study

#### 2.2.1 Patients

This study was approved by the Ethics Committee of Qilu Hospital, Shandong University, China. 78 Children who attended the pediatric outpatient clinic of Shandong University Qilu Hospital from January 2021 to December 2024 and met the enrollment criteria were included in this study. 48 children were allocated to the GFCF diet group and 30 to the control diet group. All enrolled participants were diagnosed with autism spectrum disorder (ASD) according to the Diagnostic and Statistical Manual of Mental Disorders, Fifth Edition (DSM-5). Participants were required to complete at least two 14-food intolerance IgG tests (spaced at least 6 months apart) and undergo a matched ADOS-2 or CARS assessment. Exclusion criteria included: coexisting epilepsy, inborn errors of metabolism, other neurological disorders, and receipt of alternative treatments during the testing period. In the 14-food IgG intolerance test, food-specific IgG (sIgG) levels are categorized into four grades with corresponding clinical interpretations: Grade 0 (<50 U/mL) is considered negative; Grade 1 (50–100 U/mL) signifies mild sensitivity; Grade 2 (100–200 U/mL) represents moderate sensitivity; and Grade 3 (>200 U/mL) denotes severe sensitivity.

#### 2.2.2 Grouping methodology

Through outpatient follow-up, dietary interventions implemented during the two testing periods were assessed. Eligible children with ASD were assigned to either a GFCF diet group or a Normal diet group based primarily on parental choice and clinical feasibility, constituting a non-randomized allocation.

#### 2.2.3 Statistical analysis

All data analyses were performed using SPSS 27.0 software. Baseline characteristics between the GFCF diet group and the Normal diet group were compared using t-tests or Mann–Whitney U tests. To accurately assess the intervention effect of the GFCF diet while controlling for baseline differences and regression to the mean effects, we employed a multi-strategy approach based on outcome variable types: For continuous behavioral measures (ADOS-2, CARS scores), we employed analysis of covariance (ANCOVA) or generalized linear models with gamma distribution and log-link function, using follow-up scores as the dependent variable and incorporating baseline scores as covariates. For ordered categorical measures (milk/wheat IgG antibody levels), we used ordered logistic regression models controlling for baseline levels. Additionally, to examine the dynamic pattern of intervention effects, we tested the interaction between “test diet × time” using repeated measures ANOVA and reported overall and group-specific follow-up times expressed as mean ± standard deviation. Statistical significance was set at two-tailed *p* < 0.05.

## 3 Results

### 3.1 Causal effect in forward MR analysis

**Figure 2:**
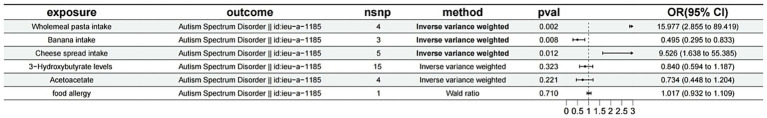
Forest plot of MR analysis exposures on genetic susceptibility to ASD.

The potential causal relationships of dietary factors, ketone bodies, and food allergy with ASD were analyzed using Mendelian randomization (MR). The comprehensive results of the MR analysis for all 199 dietary exposures, including IVW-derived ORs, 95% CIs, and *p*-values, are provided in Supplementary Table S2. The graph (Figure 2) shows that ketone bodies are not genetically related to ASD, but both ketone body ORs are less than 1(3-Hydroxybutyrate levels: OR = 0.840, 95% CI: 0.594–1.187, *p* = 0.323; Acetoacetate: OR = 0.734, 95% CI: 0.448–1.204, *p* = 0.221), suggesting that ketone bodies may exert a protective effect against ASD. However, since the *p*-value exceeds 0.05, no definitive association between exposure and outcome can be established. In addition, the ORs for food allergy were all greater than 1(OR = 1.017, 95% CI: 0.932–1.109, *p* = 0.710), suggesting that food allergy may have a promotional effect on ASD; however, because the *p* values were greater than 0.05, a significant positive association between exposure and outcome could not be concluded. Among the 199 factors analyzed, wholemeal pasta intake (OR: 15.977, 95% CI 2.855–89.419, *p* = 0.002) and cheese spread intake (OR: 9.526, 95% CI 1.638–55.385, *p* = 0.020) were positively, genetically, and causally associated with the risk of autism spectrum disorders, and banana intake (OR: 0.495, 95% CI 0.295–0.833, *p* = 0.008) was negatively, genetically, and causally associated with the risk of autism spectrum disorders (Supplementary Table S2). Cochran’s Q showed no heterogeneity (*p* ≥ 0.05) in the results of the MR analyses (Supplementary Table S3). Sensitivity analysis showed that the MR analysis results were robust (Supplementary Table S4).

### 3.2 Causal effect in reverse MR analysis

In the inverse MR analysis, we investigated the genetic correlation between ASD and Wholemeal pasta and cheese spread intake. We chose *p* < 5 × 10 ^−8^ as the threshold for significance in the study. The results showed no causal relationship between wholemeal pasta and cheese spread intake and the risk of ASD (Supplementary Table S5).

### 3.3 Result of mediation analysis

**Figure 3:**
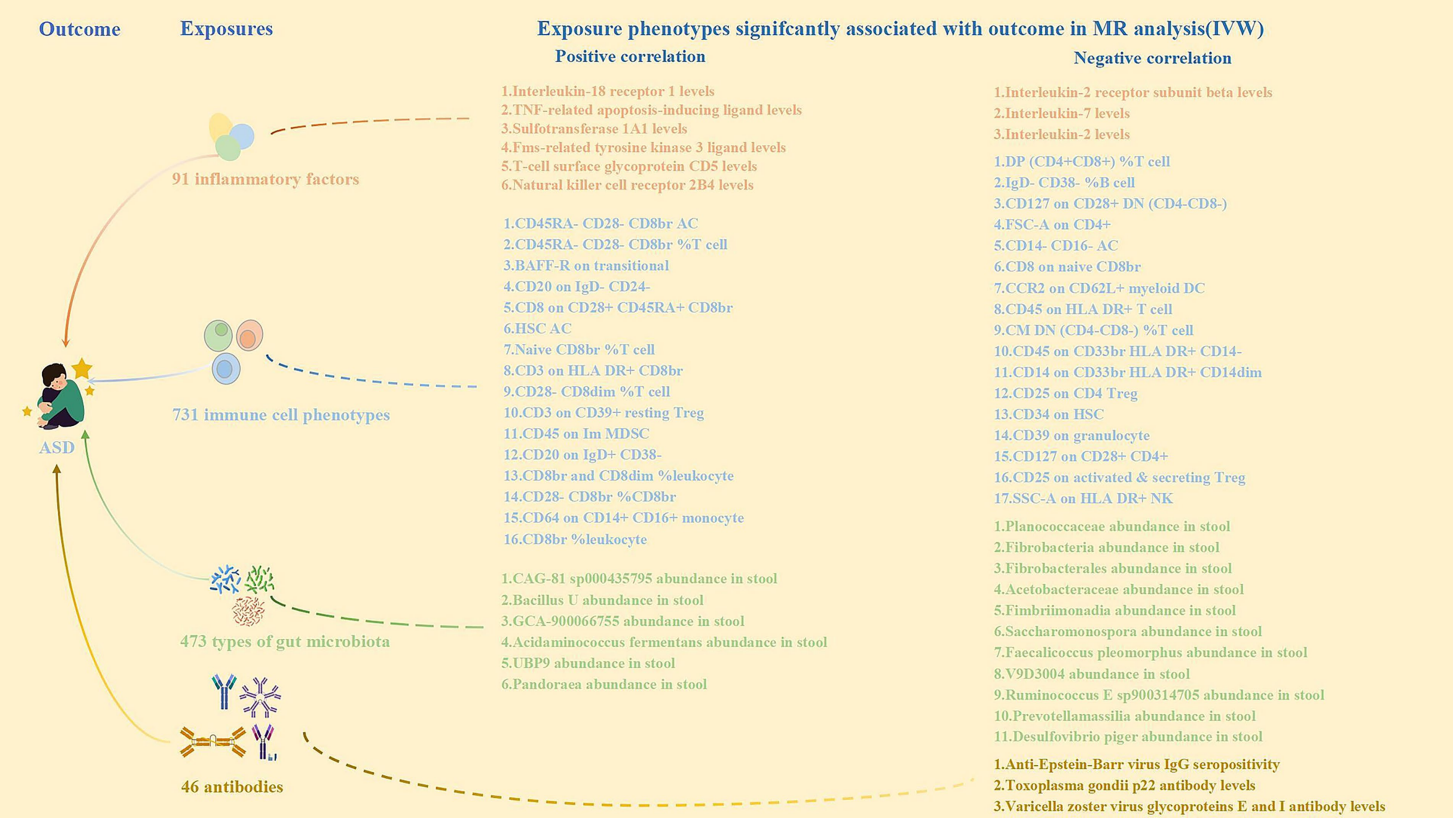
Correlation of inflammatory factors, immune cell phenotypes, types of gut microbiota and antibodies with ASD.

**Figure 4:**
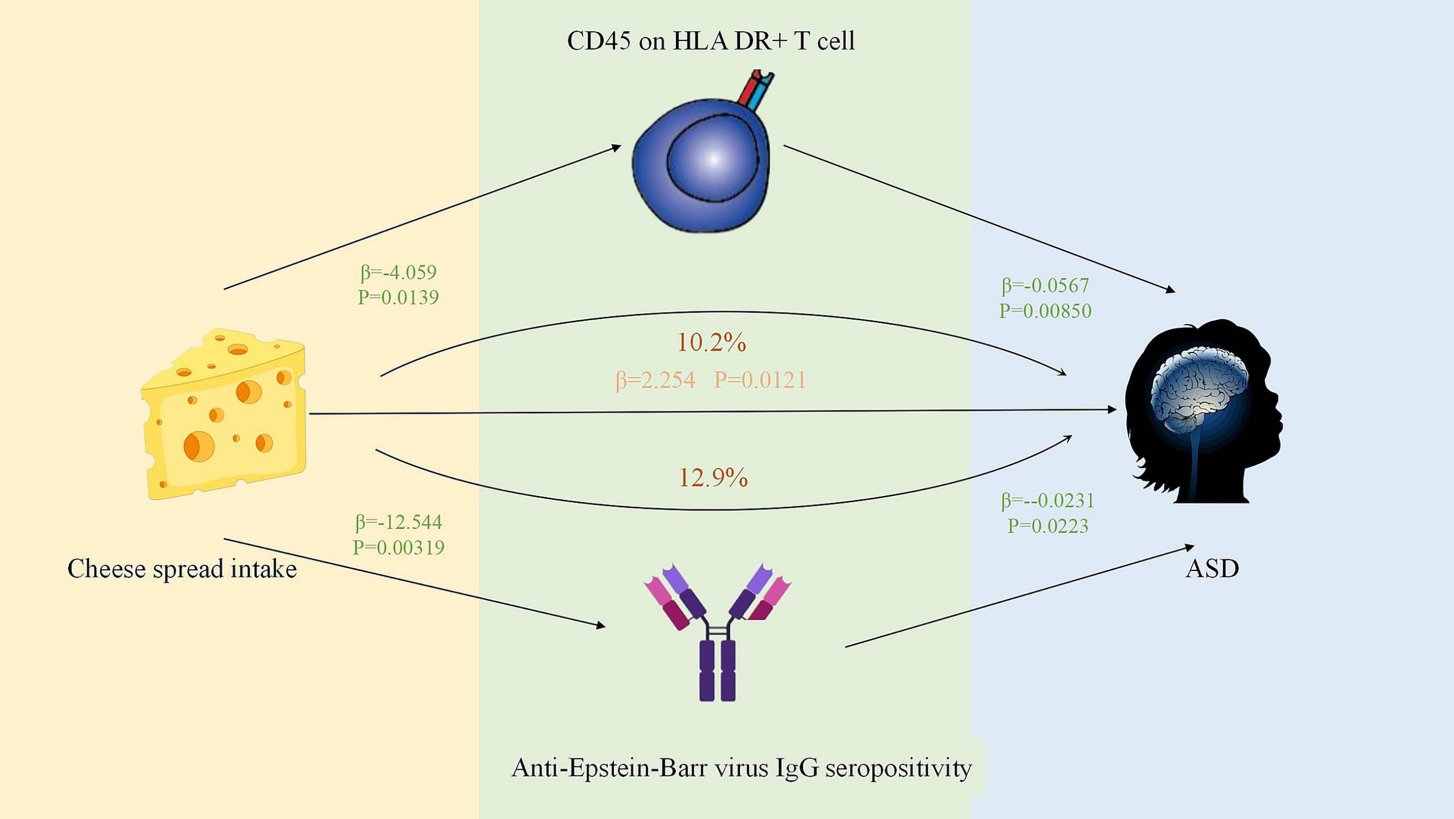
The positive mediation effect of Cheese spread intake on ASD through immune cell phenotypes and antibodies.

Based on the previously mentioned positive results, we initially analyzed the association of 91 circulating inflammatory proteins, 731 immune cells, 473 intestinal flora, and 46 antibody immunoreactivities with ASD using MR analysis (Figure 3; Supplementary Table S6). We identified nine circulating inflammatory proteins, 33 immune cells, 17 intestinal flora, and three antibody immune responses associated with ASD. We then analyzed the correlations between these nine circulating inflammatory proteins, 36 immune cells, 17 intestinal flora, and three antibody immune responses as outcome habits, and wholemeal pasta and cheese spread intake as exposure habits (Supplementary Table S7). Considering the significance and direction of action of the above MR results, the indirect effect on ASD pathogenesis was effective for cheese spread intake, mediated through CD45 on HLA DR + T cells, and anti-EBV IgG seropositivity, mediating proportions of 10.2 and 12.9%, respectively (Figure 4; Supplementary Table S8).

### 3.4 Clinical data analysis

**Figure 5:**
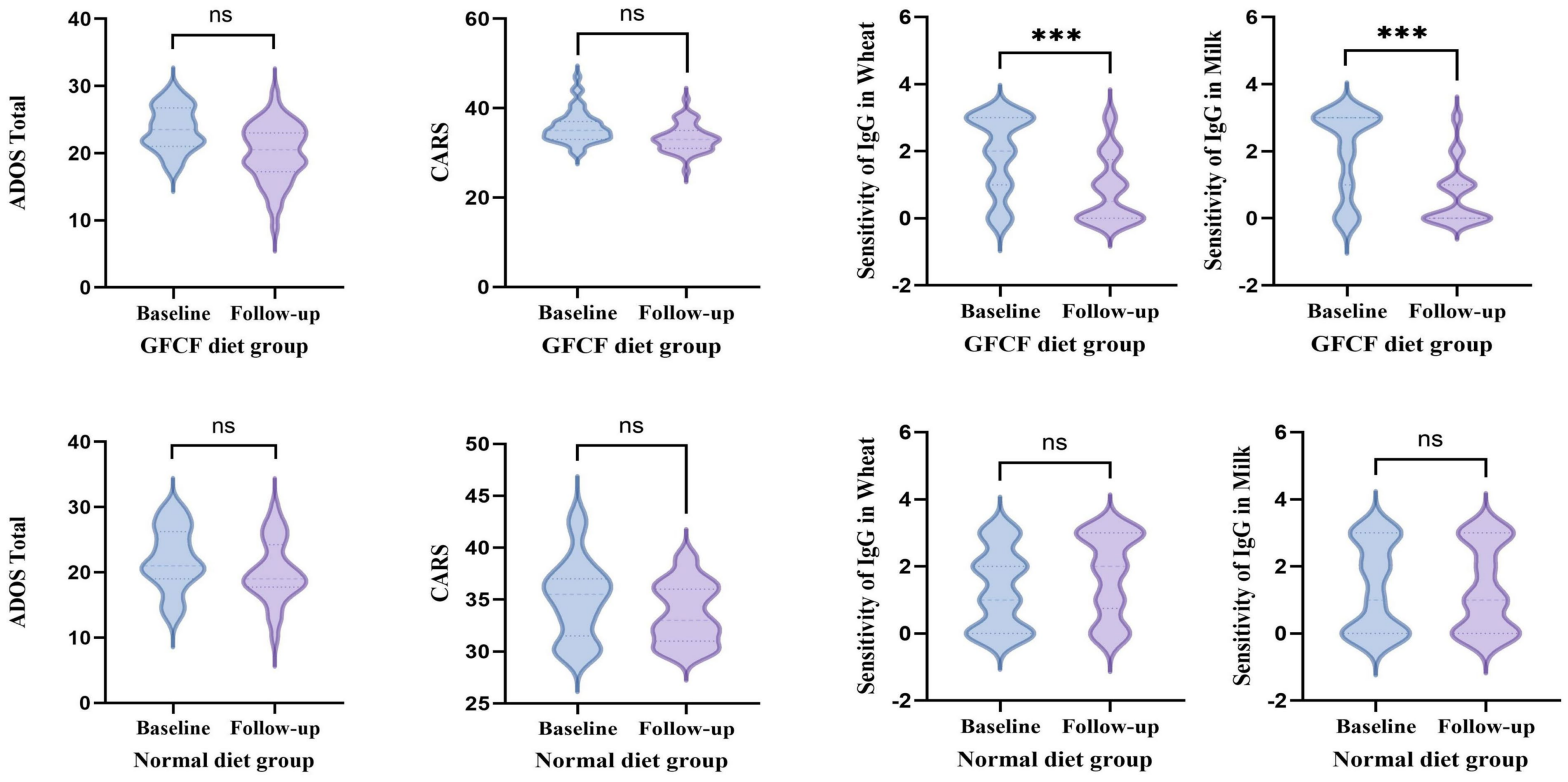
Comparison of baseline and follow-up data between the two groups.

This clinical study included 78 children with ASD. All subjects came from different families and were divided into two groups according to the grouping requirements: GFCF diet group (*n* = 48) and normal diet group (*n* = 30). Due to the low number of children with ASD who were tested for food IgG 14 items simultaneously with CARS or ADOS-2 screening, only 78 children were included in the study. The baseline characteristics of the participants are presented in Table 2 and Table 3. The two groups were comparable in terms of age and sex. To systematically evaluate the intervention effects of a GFCF diet on core autism symptoms and immune responses, this study conducted a multidimensional analysis while controlling for baseline levels. The mean follow-up duration for all patients was 9.55 months, with 8.35 ± 6.17 months for the GFCF diet group (*n* = 48) and 11.24 ± 7.45 months for the conventional diet group (n = 30). No significant difference existed between groups in follow-up duration (*p* = 0.068). Results showed no statistically significant differences in ADOS-2 or CARS scores between groups via covariance analysis or generalized linear models (ADOS-2: *F* (1,75) = 1.10, *p* = 0.298; CARS: Exp(B) = 0.981, *p* = 0.203). However, compared to improvements of 2.00 and 1.72 points in the control diet group, the GFCF diet group showed respective increases of 3.57 and 2.61 points in ADOS-2 and CARS scores. Ordered logistic regression analysis revealed a clearly significant intervention effect of the GFCF diet on milk- and wheat-specific IgG antibody levels: a significant reduction in milk-specific IgG (OR = 0.09, 95% CI 0.03–0.29, *p* < 0.001) and wheat-specific IgG antibody levels (OR = 0.06, 95% CI 0.02–0.19, *p* < 0.001). All analyses confirmed baseline levels as strong predictors of follow-up outcomes (all *p* < 0.001), supporting the necessity of treating them as covariates. Repeated measures ANOVA revealed significant main effects of time on both ADOS-2 and CARS scores [ADOS-2: *F* (1, 76) = 33.922, *p* < 0.001; CARS: *F* (1, 72) = 43.899, *p* < 0.001], while neither showed a significant “test diet × time” interaction [ADOS-2: *F* (1, 76) = 2.677, *p* = 0.106; CARS: *F* (1, 72) = 1.695, *p* = 0.197]. Conversely, milk and wheat IgG analyses revealed highly significant interactions. Both milk-specific IgG [*F* (1, 75) = 27.308, *p* < 0.001] and wheat-specific IgG [*F* (1, 76) = 39.337, *p* < 0.001] demonstrated highly significant “test diet × time” interactions (Figure 5).

**Table 2:** Baseline clinical characteristics of the 78 patients.

Characteristics	GFCF diet group (*n* = 48)	Normal diet group (*n* = 30)	*p*-value
Gender			0.404
Male	38	26	
Female	10	4	
BMI	16.99 ± 3.00	18.02 ± 4.20	0.241
Age, years	2.98 ± 1.18	2.90 ± 1.37	0.602
CARS Score	35.76 ± 3.58	35.37 ± 3.48	0.858
ADOS-2 total	23.63 ± 3.27	22.10 ± 4.78	0.131

**Table 3:** IgG sensitivity to milk and wheat between the two groups at baseline.

Clinical indicators	GFCF diet group (*n* = 48)	Normal diet group (*n* = 30)	*p*-value
Milk			0.136
Grade 0	10	12	
Grade 1	5	4	
Grade 2	6	5	
Grade 3	27	9	
Wheat			0.116
Grade 0	8	10	
Grade 1	8	6	
Grade 2	10	8	
Grade 3	22	6	

## 4 Discussion

Our two-sample MR analysis revealed the following important findings: banana intake was associated with a reduced risk of ASD development. Wholemeal pasta and cheese spread intake were associated with an elevated risk of ASD development. Mediation analysis emphasized that antibody immunity and immune cells influenced by dietary factors play key roles in the development of autism spectrum disorders. According to our analysis, CD45 on HLA DR + T cells and anti-Epstein–Barr virus IgG seropositivity had an inhibitory effect on the development of ASD, and the consumption of cheese spread reduced CD45 on HLA DR + T cells and anti-Epstein–Barr virus IgG seropositivity, thereby potentially increasing ASD risk. In addition, we also found that there is no genetic relationship between ketone bodies and ASD, but both ketone body OR values are less than 1, indicating that ketone bodies are negatively correlated with ASD, which is protective against the disease, and the food allergy OR values are greater than 1, indicating that food allergy may have a promotional effect on ASD, but the observed association lacks statistical significance, as indicated by a *p* value exceeding the 0.05 threshold, which is probably related to the insufficient sample size and thus can only be used as a reference. Our clinical research indicates that a GFCF diet significantly reduces milk/wheat-specific IgG antibody levels in children with ASD, while potentially exerting positive effects on core symptoms. Although this diet has not yet demonstrated statistically significant superiority in symptom relief, its capacity to effectively modulate food-specific immune responses highlights its potential value in the field of immune intervention. Data indicate that compared to the normal diet group, the GFCF diet group demonstrated a significantly superior trend toward improvement in reducing food-specific antibody levels. This finding suggests that the GFCF dietary regimen may have potential clinical applications and provide new perspectives for the treatment of ASD. However, due to the relatively small sample size of the current study, to more comprehensively and accurately assess the efficacy and applicability of the GFCF diet, in-depth multicenter large-sample studies are urgently needed for further validation. These findings emphasize the importance of specific dietary patterns in the treatment of autism and provide valuable evidence for the general public.

An increasing amount of research indicates that the gut-brain axis is crucial in the development of ASD. Emerging evidence indicates that gut microbiota modulates central neurological activities through integrated neuroendocrine-immune signaling mechanisms, thereby contributing to neurodevelopmental pathophysiology in conditions such as ASD and the generation of microbial metabolites (28, 29). Studies have found altered gut microbiota in ASD, a condition referred to as dysbiosis (30). Gut dysbiosis leads to systemic inflammation and neuroinflammation, which impairs brain function (gut-brain axis) (31). An example is the effect of GFCF diets on ASD (15, 32). The implementation of GFCF diets is grounded in the “opioid excess theory.” This theory suggests that specific food proteins can be broken down into their constituent, opioid peptides. These peptides can enter the bloodstream and affect the central nervous system. Consequently, diets that limit protein intake, specifically GFCF diets, are believed to help alleviate the behavioral symptoms in children with autism.

Wholemeal pasta and cheese spread intake belonged to the gluten and casein diets, respectively. We found that wholemeal pasta and cheese spread intake increased genetic susceptibility to ASD, as determined by Mendelian randomization analysis. There is a proposition that gluten-free and casein-free (GFCF) diets might help ease the symptoms of autism spectrum disorders (ASD) and support the neurodevelopment of children with ASD. However, existing studies have not yet reached a definitive conclusion (33–35).

One potential mechanism through which GFCF diets might impact ASD is explained by the opioid excess theory. According to this theory, milk and gluten/gelatin proteins activate the immune system in the gastrointestinal tract. This inflammatory cascade triggers cytokine secretion and mediator release, thereby compromising the integrity of the intestinal epithelial barrier via enhanced paracellular permeability. Additionally, these proteins can alter the gut microbiota. Alterations in gut microbiota composition may compromise intestinal barrier function through dysregulation of tight junction protein expression and localization, including critical structural components such as connexins. Consequently, diet-derived proteins, including milk constituents and gluten/gelatin compounds, can undergo intestinal translocation into the systemic circulation and induce immune responses. Subsequent passage across a compromised blood–brain barrier enables access to the central nervous system (CNS) parenchyma. Within the CNS, the breakdown of casein and gliadin releases opioid-like peptide. These neuroactive peptides may impair cellular antioxidant defenses within neuronal populations, potentially exacerbating the core behavioral manifestations of ASD through redox imbalance mechanisms. Moreover, Gluten and casein are prevalent dietary allergens capable of triggering inflammatory cascades and inducing specific IgA/IgG antibody-mediated humoral immune responses, particularly in susceptible individuals. Additionally, research has revealed that ASD often exhibits deficiencies in ileal transcripts that encode disaccharidases and hexose transporter proteins. These proteins play a crucial role in the digestion and transport of carbohydrates within enterocytes, and their deficiencies can result in difficulties in lactose digestion from milk (36). Pennesi et al. (37) documented significant behavioral improvements in ASD following GFCF dietary intervention, with notable alleviation of gastrointestinal manifestations—particularly constipation and diarrhea—and enhanced physiological outcomes specifically within the allergy-symptomatic subgroup. However, the current methodological consensus indicates a lack of robust evidence supporting GFCF dietary interventions as efficacious ASD therapeutics, necessitating further investigation through large-scale randomized controlled trials incorporating double-blind protocols (16, 38).

It has now been shown that there is a strong relationship between food allergy (FA) and ASD. Food allergy is an abnormal or overpowering immune response to food proteins that can be mediated by IgE or non-IgE. Epidemiological investigations have found that the prevalence of both ASD and FA has increased significantly in recent decades, suggesting a possible association between the two. A current study has shown that children with ASD are more likely to develop gastrointestinal symptoms than normal children (14, 39), and that these symptoms are strongly associated with the severity of autistic behavior (40). Avoidance of certain foods, such as bran protein and gluten, not only relieves gastrointestinal symptoms but also improves the cognitive abilities of children with ASD. Food allergy, a common cause of gastrointestinal reactions, is closely associated with ASD. Emerging evidence implicates dysregulated neuroimmune interactions, mast cell activation, gut microbiota perturbations, and compromised intestinal barrier integrity as potential synergistic contributors to the pathogenesis of FA and ASD.

Originally developed as a therapeutic intervention for refractory epilepsy, the ketogenic dietary regimen is an established clinical approach for managing seizure disorders (41). Clinical evidence indicates that antiepileptic medications may ameliorate core autistic manifestations in patients with epilepsy-comorbid ASD. This therapeutic response implies shared pathophysiological mechanisms between seizure disorders and autism spectrum disorder (42). In one study, children with ASD were treated with ketogenic diet therapy, and the results showed that both CARS and ASC scale scores were significantly lower than those of the control group, while the core symptoms of the children were also significantly improved. One mechanism by which ketogenic diet therapy may affect ASD is the modulation of neurotransmitter levels. In recent years, many scholars have suggested that excitatory/inhibitory neurotransmitter imbalance is an important pathogenesis of ASD. Some studies have found that certain aminobutyric acid receptor subtypes are associated with autism (43, 44). As the brain’s primary inhibitory neurotransmitter, gamma-aminobutyric acid (GABA) deficiency may contribute to the pathogenesis of seizure disorders and autism spectrum disorder (ASD) through disrupted neuronal excitability regulation. Some studies have shown that ketone bodies may elevate central nervous system GABA concentrations, thereby improving the symptoms of autism. Veenstra et al. (45) used MRI spectroscopy to confirm that some children on ketogenic diets had significantly higher brain GABA concentrations, suggesting that ketogenic diets may improve the symptoms of epilepsy and ASD by affecting the metabolic levels of central neurotransmitters.

Through Mendelian analysis, we found that bananas have a potential protective effect against ASD. Bananas are a nutritionally dense fruit source that provides tryptophan, an essential amino acid that serves as a metabolic precursor for serotonin biosynthesis. This neurotransmitter pathway contributes to the regulation of the affective state. In children with autism, which is often associated with impaired emotion regulation, appropriate supplementation with tryptophan-containing foods may help alleviate these symptoms (46). In addition, bananas are rich in dietary fiber, which can promote intestinal peristalsis and prevent constipation, which is also beneficial for the intestinal health of children with autism. However, no study has demonstrated a protective effect of bananas against autism. However, it is important to note that while bananas may have these potential benefits for children with autism, they do not have a direct therapeutic effect on autism. Bananas are listed as foods that are high in phenols. Oxidative stress occurs when the production of reactive oxygen species (ROS) exceeds the body’s capacity to eliminate them, especially due to impairments in the antioxidant defense system. Given that oxidative stress is implicated in the development of ASD, disruptions in the antioxidant defense system can result in changes in neuronal structure and overall brain function, as well as inflammation and immune dysfunction. Foods rich in antioxidants may have potential therapeutic benefits for individuals with autism (47). Accumulating evidence indicates that antioxidant supplementation (e.g., polyphenols and flavonoids) may ameliorate core autistic manifestations. Recent mechanistic insights have revealed that dietary polyphenols undergo microbial biotransformation within the gut microbiota, generating bioactive metabolites with enhanced antioxidant potency and neuroprotective efficacy (48). Polyphenols are converted into bioaccessible phenolic metabolites through microbial biotransformation in the gut. These compounds subsequently enter the systemic circulation and traverse the blood–brain barrier to exert neuroactive properties within the central nervous system compartments (49). Thus, polyphenols act as natural antioxidants and may improve ASD symptoms.

These dietary therapies may improve ASD symptoms by correcting gastrointestinal inflammation or allergy, energy metabolism disorders, oxidative stress damage, neurotransmitter disorders, and intestinal flora imbalances in patients with ASD. Nonetheless, ASD management necessitates a multidisciplinary intervention strategy incorporating behavioral, speech-language, and occupational therapies. Adjunctive pharmacotherapy may be indicated to address comorbid symptom complexes in select clinical presentations. Dietary modifications may help support the overall health of individuals with ASD but are not a direct treatment for ASD. A healthcare professional should be consulted before making any dietary changes.

Several studies have analyzed the causal relationship between immune cells, inflammatory factors, intestinal flora, and ASD using Mendelian randomization methods; however, no study has analyzed the genetic correlation between antibody immunity and ASD. Here, we discuss the relationship between antibody immunity and ASD. Substantial epidemiological evidence links autism spectrum disorder (ASD) to diverse infectious agents, including human herpesviruses and Toxoplasma gondii. While multiple studies have confirmed the association between perinatal pathogen exposure and ASD risk, the etiological contributions remain mechanistically unestablished. Therefore, elucidating these causal pathways is critical for developing targeted preventive strategies and precision therapeutics for this neurodevelopmental disorder. In this study, we used Mendelian randomization to explore the potential relationship between antibody-mediated immune responses to infectious disease pathogens and ASD risk. Our results showed that Varicella zoster virus glycoproteins E and I antibody levels, Anti-Epstein–Barr virus IgG seropositivity, Toxoplasma gondii p22 antibody levels were associated with a reduced risk of ASD. Seropositivity for varicella-zoster virus (VZV) IgG and glycoprotein E and I antibodies typically indicates acquired immunity against primary VZV infections. However, emerging epidemiological evidence suggests that latent VZV infection may be a risk factor for neurodevelopmental disorders and certain neurodegenerative conditions (50). Maternal immune responses to herpes simplex virus-2 (HSV-2) have been implicated in the elevated susceptibility to autism spectrum disorder (ASD) in offspring. Conversely, clinical interventions, including herpes zoster vaccination and targeted antiviral regimens, have been shown to be effective in mitigating dementia risk by modulating shared neurodegenerative pathways (51, 52). Attempts have been made to use herpes zoster vaccination and antiviral therapy in the treatment of ASD. Thus, effective virological control of active herpesvirus infections is more important for mitigating ASD risk than achieving seropositivity status alone. This paradigm underscores VZV vaccination as a strategic intervention for suppressing the viral reactivation pathways implicated in ASD pathogenesis. Mendelian randomization analyses revealed an inverse association between elevated Toxoplasma gondii p22 seropositivity and ASD susceptibility, suggesting the potential protective effects of anti-parasitic immune responses. Toxoplasma gondii, an obligate intracellular protozoan parasite, exhibits neurotropic properties enabling central nervous system (CNS) invasion. This pathogen poses significant clinical risks, particularly severe neuropsychiatric sequelae in immunocompromised patients (53). P22 is the main immunogenic antigen of Toxoplasma gondii. Toxoplasma gondii p22 antibody level is the concentration of antibodies specific to the Toxoplasma gondii p22 protein. Current epidemiological evidence has failed to establish a definitive association between Toxoplasma gondii infection and ASD in pediatric populations. However, emerging mechanistic hypotheses propose that neurochemical alterations and structural brain anomalies observed in ASD may parallel the pathophysiological features of chronic toxoplasmosis, warranting further investigation (54). Spann et al. (55) documented elevated maternal Toxoplasma gondii IgM seropositivity correlating with diminished likelihood of ASD offspring. Conversely, maternal IgG seropositivity was inversely associated with ASD risk. The authors postulated two immunological mechanisms: parasite-directed immune activation or systemic immune cascade initiation may underlie this phenomenon, necessitating further mechanistic investigation. Epstein–Barr Virus (EBV) has been associated with autoimmune diseases and a variety of neoplasms, potentially increasing the risk of autoimmune diseases. However, it has been shown that different EBV antibodies have different effects on autoimmune diseases (56). Although some antibodies increase the risk of disease, most have protective effects. Mendelian randomization analyses have identified a protective association between anti-Epstein–Barr virus (EBV) IgG seropositivity and autoimmune outcomes, potentially mediated by temporal variations in humoral immune response kinetics following primary EBV exposure.

These findings provide strong evidence to support the early prevention and dietary management of these disorders. Future studies should continue to investigate these associations and consider the interaction of different dietary settings to further elucidate the mechanisms and inform dietary guidelines aimed at dietary autism.

Our current research using genetic tools strongly suggests that higher intakes of two specific foods—wholemeal pasta (as a gluten-rich source) and cheese spread (as a casein-rich source) may be risk factors for ASD. Clinical studies suggest that implementing GFCF diets appears to improve symptoms in individuals with ASD. However, our study had several limitations. First, we examined genetic correlations only for wholemeal bread and cheese spread, excluding other gluten- or casein-containing food. Second, the study’s primary focus on individuals of European ancestry constrains the generalizability of these findings to populations with distinct demographic characteristics, necessitating future validation in diverse ethnic groups. Third, the sample sizes of the public databases used in this study were relatively small, potentially leading to weaker overall statistical significance. Fourth, our study had a small sample size, and further validation is needed in a multicenter large-sample study. Collectively, while Mendelian randomization analyses reveal putative causal pathways at the molecular level, subsequent experimental validation remains imperative to authenticate physiological impacts and delineate the pathophysiological processes underlying diet-associated disorders. Subsequent genome-wide association studies are expected to utilize the expanded cohort to identify key genetic variants that will provide important insights into the early prevention of related diseases.

## 5 Conclusion

In conclusion, our study suggests that the intake of wholemeal pasta and cheese spread is a risk factor for ASD. These findings may help clinicians enhance health education for patients with ASD and encourage them to change their dietary factors. The results of our MR and mediation analyses suggest that wholemeal pasta and cheese spread intake increases the risk of ASD development. CD45 on HLA-DR + T cells and anti-Epstein–Barr virus IgG seropositivity played a role in the relationship between the two mediates this relationship. Furthermore, while some clinical observations suggest that ketogenic diets and allergen avoidance might ameliorate ASD symptoms, our MR analysis did not support a significant association between ketone bodies and ASD risk, necessitating further confirmation of these findings. This study will help guide ASD treatments. In addition, we found a significant negative correlation between antibody-mediated immune responses against infectious agents, such as Varicella zoster virus glycoproteins E and I antibody levels, Anti-Epstein–Barr virus IgG seropositivity, Toxoplasma gondii p22 antibody levels and ASD risk. These findings highlight the complexity of immune system responses and set the stage for further exploration of the role of immunomodulation in the pathogenesis of ASD. Although this study provides important insights, more extensive epidemiological and experimental studies are needed to validate these findings.

## Data Availability

The raw data supporting the conclusions of this article will be made available by the authors, without undue reservation.
